# Supplementary material for: A Comparative Effect of 12-Week Dietary Intervention of Policosanol (Raydel®) and Red Yeast Rice (RYR, Kobayashi) in Managing Dyslipidemia and Organ Damage in Hyperlipidemic Zebrafish
Source: Pharmaceuticals (Basel). 2025 Feb 1;18(2):200. doi: 10.3390/ph18020200 (PMC11859080; doi:10.3390/ph18020200)
Supplement: Supplementary file 1 [file pharmaceuticals-18-00200-s001.zip › pharmaceuticals-3460469-supplementary.pdf]

Supplementary Materials

Section S1-Materials

1. List of the used chemicals: Dihydroethidium (DHE, 104821-25-2, Cat #37291), and acridine orange (AO, 65-61-2, Cat#A9231), oil red O (Cat#O0625), and 2-phenoxyethanol (Sigma P1126; St. Louis, MO, USA), paraoxon-ethyl (Cat. No. 36186) and 5-bromo-4-chloro-3-indolyl β D-galactopyranoside (X-gal, Cat#B54252) were procured from Sigma–Aldrich (St. Louis, MO, USA). All other chemicals and reagents else otherwise stated were of analytical grade and used as supplied.

Section S2-Methods

2. Analysis of plasma

Blood (2 μL) was drawn from the hearts of the adult fish, combined with 3 μL of phosphate-buffered saline (PBS)-ethylenediaminetetraacetic acid (EDTA, final concentration, 1 mM) and then collected in EDTA-treated tubes. The plasma total cholesterol (TC) and triglyceride (TG) were determined using commercial assay kits (cholesterol, T-CHO, and TGs, Cleantech TS-S; Wako Pure Chemical, Osaka, Japan) as per the method suggested by the suppliers. In brief, 5 μL serum was mixed with 200 μL reaction mixture (supplied with a commercial assay kit) for the TC analysis. The content was incubated at 37°C for 10 min, resulting in a red-colored product quantified by adsorption at 490 nm (Microplate reader, Bio-Rad, Hercules, CA, USA).

Similarly, 5 μL serum was mixed with a 200 μL TG-specific reaction mixture (supplied with a commercial assay kit) for TG analysis. The content was incubated for 10 min at 37°C, and the formed colored product was quantified by taking adsorption at 490 nm.

For HDL-C analysis, serum was mixed in an equal ratio with the separation solution (supplied with a commercial assay kit), followed by centrifugation at 3,000 rpm for 10 min. The supernatant (20 μL) was collected and blended with a 200 μL reaction mixture (supplied with a commercial assay kit). After 10 min incubation at 37°C, red color intensity corresponding to HDL-C was quantified by taking absorption at 490 nm.

The commercial diagnostic kit (Asan Pharmaceutical, Hwasung, Republic of Korea) was used to quantify aspartate transaminase (AST) and alanine transaminase (ALT) levels in the serum, following the instructions suggested by the manufacturers. Briefly, 5 μL of serum was combined with 250 μL of either AST or ALT-specific solution, as supplied in the diagnostic kit. Following a 30 min incubation for AST or 60 min incubation of ALT at 37°C, the mixture was then blended with 250 μL of the respective coloring reagent (AST or ATL-specific, provided in the diagnostic kit). After a subsequent 20 min incubation at RT, 250 μL of 0.4 N NaOH was introduced to halt the reaction. Finally, the AST and ATL were quantified by measuring absorbance at 490 nm.
